# Auto-correlation in the motor/imaginary human EEG signals: A vision about the *F_DFA_* fluctuations

**DOI:** 10.1371/journal.pone.0183121

**Published:** 2017-09-14

**Authors:** Gilney Figueira Zebende, Florêncio Mendes Oliveira Filho, Juan Alberto Leyva Cruz

**Affiliations:** 1 Department of Physics, State University of Feira de Santana, Bahia, Brazil; 2 Gilberto Gil Campus, Estácio de Sá University, Bahia, Brazil; 3 Computational Modeling Program, SENAI CIMATEC, Bahia, Brazil; University of Electronic Science and Technology of China, CHINA

## Abstract

In this paper we analyzed, by the *F*_*DFA*_ root mean square fluctuation (rms) function, the motor/imaginary human activity produced by a 64-channel electroencephalography (EEG). We utilized the Physionet on-line databank, a publicly available database of human EEG signals, as a standardized reference database for this study. Herein, we report the use of detrended fluctuation analysis (DFA) method for EEG analysis. We show that the complex time series of the EEG exhibits characteristic fluctuations depending on the analyzed channel in the scalp-recorded EEG. In order to demonstrate the effectiveness of the proposed technique, we analyzed four distinct channels represented here by *F*_3_32, *F*_6_37 (frontal region of the head) and *P*_3_49, *P*_6_54 (parietal region of the head). We verified that the amplitude of the *F*_*DFA*_ rms function is greater for the frontal channels than for the parietal. To tabulate this information in a better way, we define and calculate the difference between *F*_*DFA*_ (in *log* scale) for the channels, thus defining a new path for analysis of EEG signals. Finally, related to the studied EEG signals, we obtain the auto-correlation exponent, *α*_*DFA*_ by DFA method, that reveals self-affinity at specific time scale. Our results shows that this strategy can be applied to study the human brain activity in EEG processing.

## Introduction

**Fig 1:**
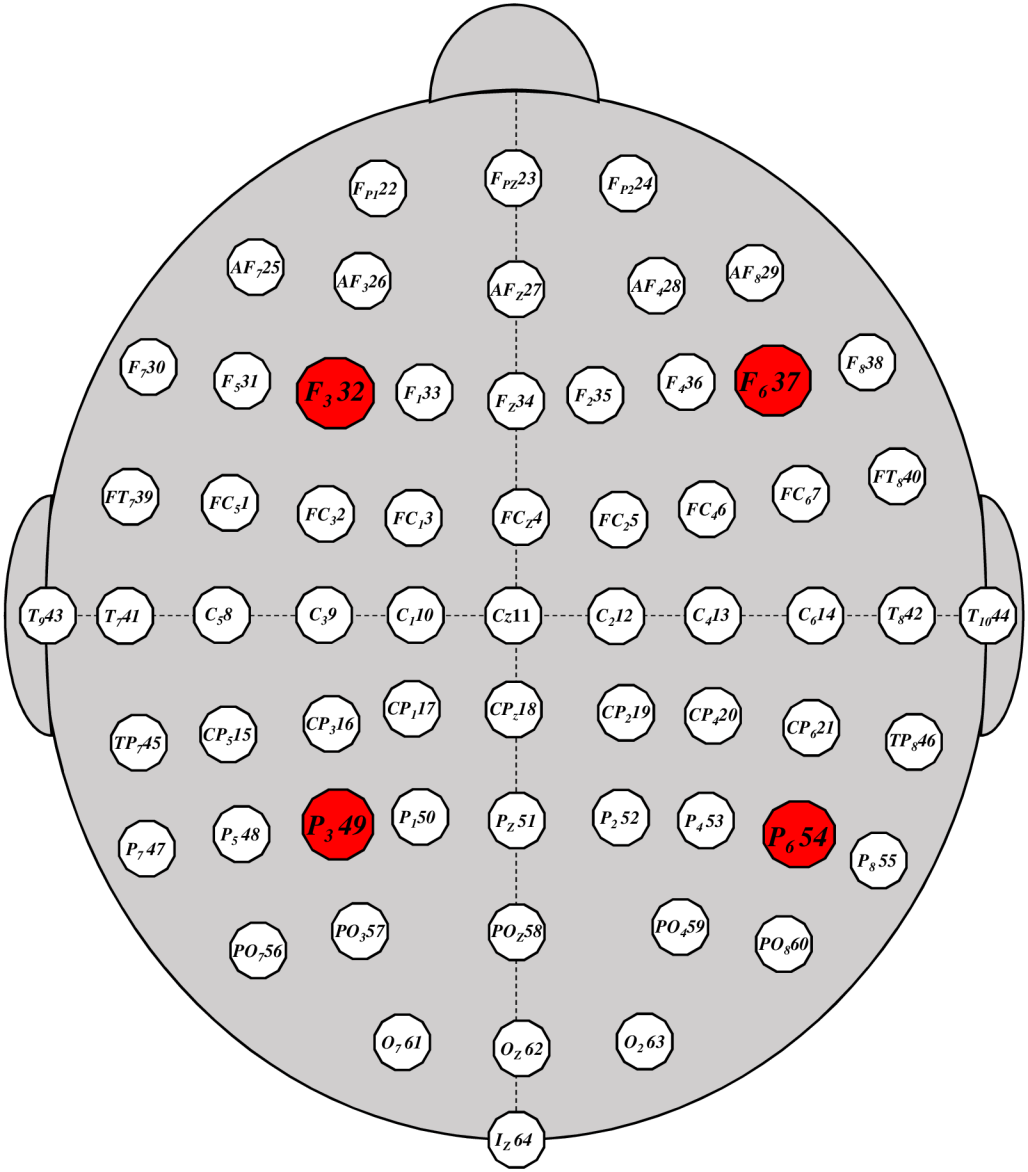
(Color on-line) Setup for EEG channels: 64 electrodes as per the international 10-20 system (excluding electrodes Nz, F9, F10, FT9, FT10, A1, A2, TP9, TP10, P9, and P10). The specific points (full circle in red) (*F*_3_32, *F*_6_37, *P*_3_49, *P*_6_54) identify the channels used in this paper.

The electroencephalogram (EEG) is generally an noninvasive method to record electrical activity of the brain. EEG machine is composed of electrodes, which are placed on the scalp to detect the brain waves [1]. Most EEG machines amplify the signals and records on computer by European Data Format (EDF) file. The EEG measurement is the voltage fluctuations, and with this measure it is possible to diagnose tumors, stroke, epilepsy, and other brain disorders which leads to some abnormalities in EEG readings. Despite the spatial resolution limitations, EEG remains a valuable tool for research and diagnosis, especially when a time resolution interval of milliseconds is required (which is not possible with computed tomography or magnetic resonance imaging) [2, 3]. See [4] for history of EEG. Therefore, in the last two decades, emerges the field of Brain-Computer Interface (BCI) [5], providing communication and control capabilities to people with severe motor inability. Hence, the typical BCI system is built for one particular method and is not adjusted to others. In view of this limitation [6, 7] implemented a platform called BCI2000 [8], a general-purpose software system for BCI research. Thus, from BCI2000 system and the full documentation presented in [9], we download and analyzed, as we will see below, data of subjects performed different motor/imagery Tasks in 64-channel EEG, Fig 1.

Here, each subject performed 14 experimental runs: two one-minute baseline (one with eyes open, one with eyes closed), and three two-minute of the following Tasks [9]:

- **Task 1** a target appears on either the left or the right side of the screen. The subject opens and closes the corresponding fist until the target disappears. Then the subject relaxes. (Real (L/R));
- **Task 2** a target appears on either the left or the right side of the screen. The subject imagines opening and closing the corresponding fist until the target disappears. Then the subject relaxes. (Imag (L/R));
- **Task 3** a target appears on either the top or the bottom of the screen. The subject opens and closes either both fists (if the target is on top) or both feet (if the target is on the bottom) until the target disappears. Then the subject relaxes. (Real (T/D));
- **Task 4** a target appears on either the top or the bottom of the screen. The subject imagines opening and closing either both fists (if the target is on top) or both feet (if the target is on the bottom) until the target disappears. Then the subject relaxes. (Imag (T/D)).

In summary, see Table 1:

**Table 1:** 14 experimental runs for each subject: Two one-minute baseline (eyes open/closed) and three two-minute of four Tasks.

1 eyes open	2 eyes closed	-	-
**3** Task 1	**4** Task 2	**5** Task 3	**6** Task 4
**7** Task 1	**8** Task 2	**9** Task 3	**10** Task 4
**11** Task 1	**12** Task 2	**13** Task 3	**14** Task 4

Based on these Tasks and given that usually the diagnostic of EEG focus on the spectral content, like a Fourier analysis, we analyzed the brain activity of 10 subjects in three experiments, by DFA method, randomly chosen in [9]. Our focus was only in four channels, represented in the Fig 1, by specific points (full circle in red) in the brain: (i) *F*_3_32 located in the frontal region, left hemisphere; (ii) *F*_6_37 located in the frontal region, right hemisphere; (iii) *P*_3_49 located in the parietal region, left hemisphere; (iv) *P*_6_54 located in the parietal region, right hemisphere. These points were selected because, as you know, the left side of the brain is responsible for controlling the right side of the body, and performs tasks that have to do with logic. On the other hand, the right hemisphere coordinates the left side of the body, and performs tasks that have do with creativity. Already the parietal lobe, integrates sensory information, including spacial sense and navigation [10, 11]. Thus, we can cross the four channels (hemispheres) in attempt to analyze the EEG, by *F*_*DFA*_ rms function and the *α*_*DFA*_ exponent. This is a new methodology of EEG analysis, where interesting results can be seen and easily applicable in subsequent studies, as we will see below.

## Materials and methods

### 0.1 Database

**Fig 2:**
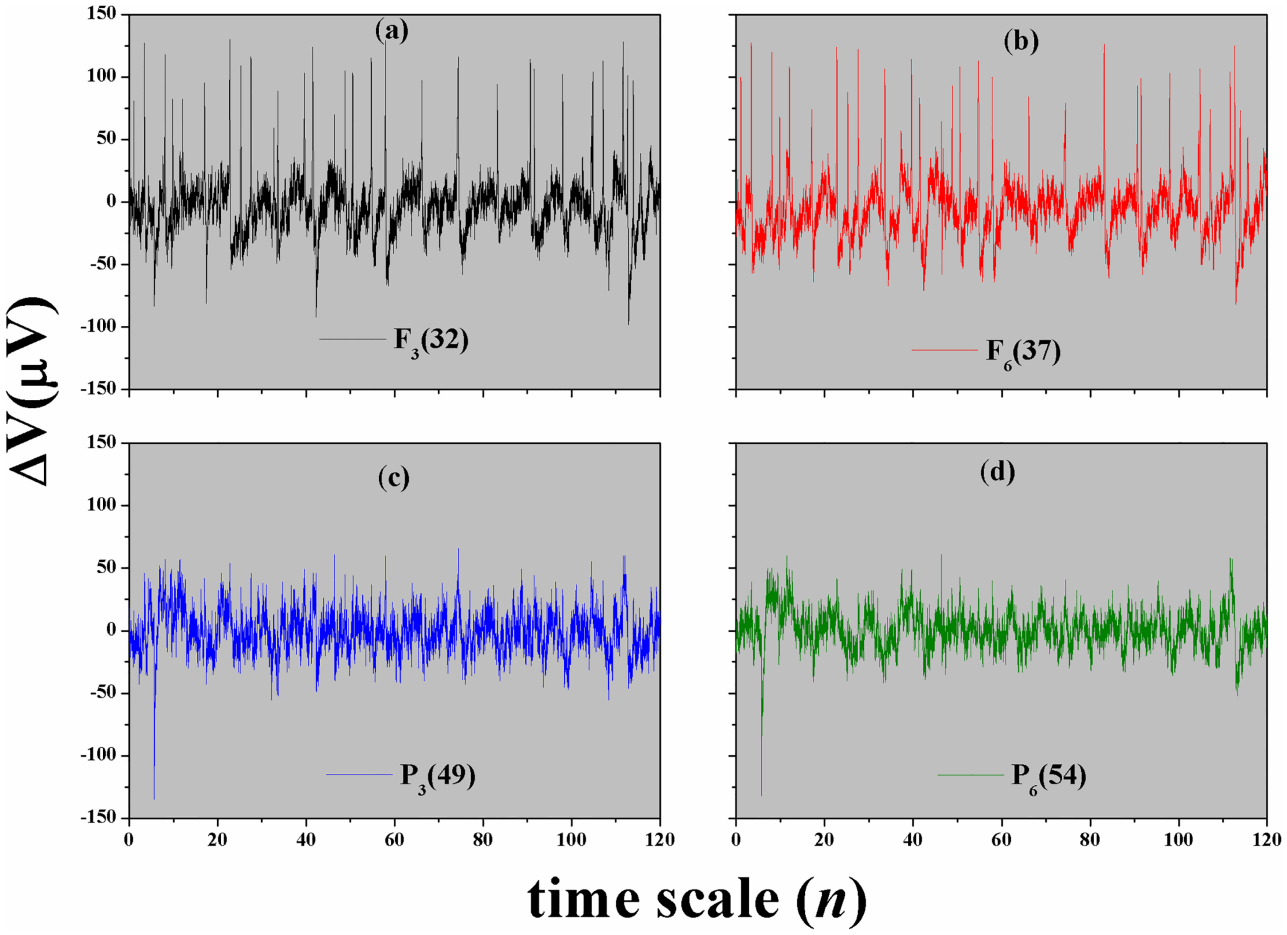
(Color on-line) Original time series of the EEG signal. Channels (a) *F*_3_32 and (b) *F*_6_37 represent the frontal region of the brain, and (c) *P*_3_49 and (d) *P*_6_54 represent the parietal region. These time series correspond to the subject *S*020 at the Task 1 (open and close left or right fist).

The time series were analyzed by DFA method from the database available in https://physionet.org/pn4/eegmmidb/. We selected randomly ten subjects in this database, that are: *S*020, *S*029, *S*043, *S*046, *S*050, *S*051, *S*060, *S*071, *S*086, and *S*099. Each subject performed three experiments for a defined Task (see Table 1). The data are provided in EDF+ format (containing 64 EEG signals, at 160 samples per second, and an annotation channel). The variable measured by the EEG device is the electrical voltage on scalp, with amplitude quite small in units of microvolts (*μV*). Fig 2 presents an example of these time series for the channels *F*_3_32, *F*_6_37, *P*_3_49, and *P*_6_54.

### 0.2 DFA method

In order to analyze the EEG time series, we briefly present the DFA method [12], which involves the following steps:

1. Consider a correlated signal intensity, *u*(*i*) (EEG signal), where *i* = 1, …, *N*_*max*_, and *N*_*max*_ is the total number of points in the time series. We integrate the signal *u*(*i*) and obtain $y\left(k\right)=\sum_{i=1}^{k}\left[u\left(i\right)-<u>\right]$, where <*u*> is the average of *u*;
2. The integrated signal *y*(*k*) is divided into boxes of equal length *n* (time scale);
3. For each *n*-size box, we fit *y*(*k*), using a polynomial function of order *l*, which represents the trend in the box. The *y* coordinate of the fitting line in each box is denoted by *y*_*n*_(*k*), since we use a polynomial fitting of order *l*, we denote the algorithm as DFA-*l*;
4. The integrated signal *y*(*k*) is detrended by subtracting the local trend *y*_*n*_(*k*) in each box (of length *n*);
5. For a given *n*-size box, the *F*_*DFA*_(*n*) root mean square fluctuation (rms) function for this integrated and detrended signal is given by
  $$\begin{array}{c} F_{DFA}\left(n\right)=\sqrt{\frac{1}{N_{max}}\sum_{k=1}^{N_{max}}{\left[y\left(k\right)-y_{n}\left(k\right)\right]}^{2}}; \end{array}$$ (1)
6. The above computation is repeated for a broad range of scales (*n*-sizes box) to provide a relationship between *F*_*DFA*_(*n*) and the box size *n*, characterized by a power-law $F\left(n\right)∼n^{\alpha_{DFA}}$. In this way, *α*_*DFA*_ is the scaling exponent, a self-affinity parameter representing the long-range power-law correlation properties of the signal, such as [13], see Table 2:

**Table 2:** Information about DFA exponent.

exponent	type of signal
*α*_*DFA*_ < 0.5	anti-persistent
*α*_*DFA*_ ≃ 0.5	uncorrelated, white noise
*α*_*DFA*_ > 0.5	long-range correlated persistent
*α*_*DFA*_ ≃ 1	1/*f* noise
*α*_*DFA*_ > 1	non-stationary
*α*_*DFA*_ ≃ 3/2	Brownian noise

The advantages of DFA over many others methods are that it permits the detection of long-range correlations embedded in seemingly non-stationary time series, and also avoids the spurious detection of apparent long-range correlations, that are an artifact of non-stationarity [14, 15]. The obtained exponent is similar to the Hurst exponent [16], except that DFA may also be applied to signals whose underlying statistics (such as mean and variance) or dynamics are non-stationary [17–20]. See the papers [19, 21–24], for which DFA and EEG were applied.

**Fig 3:**
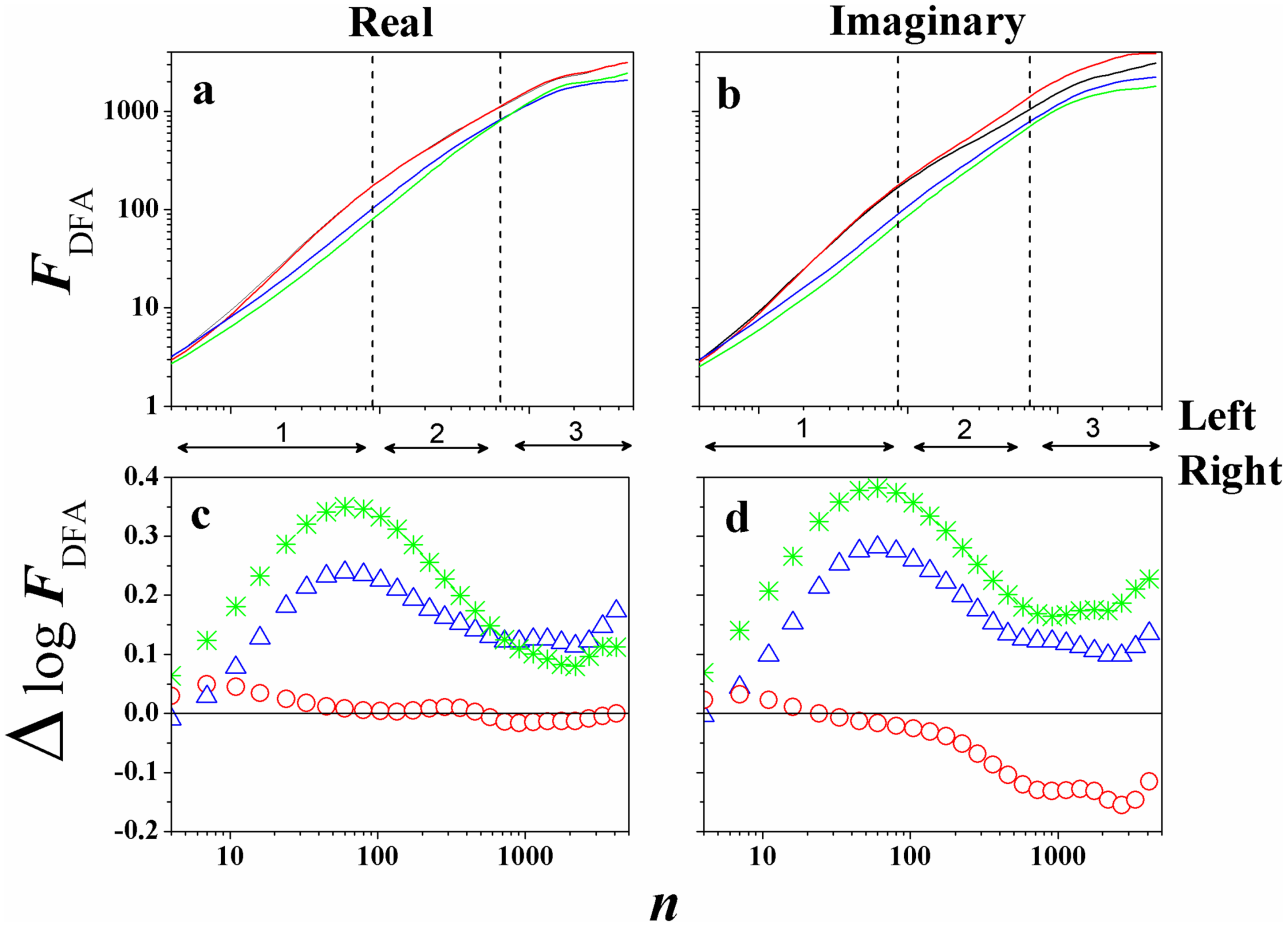
(Color on-line) *F*_*DFA*_ in function of *n* for *S*020 experiment 1 for (Left/Right): (a) Real and (b) Imaginary case. Black line represents *F*_3_32, red line *F*_6_37, blue line *P*_3_49, and green line *P*_6_54. Also, the figures below show the difference Δ*logF*_32;*xx*_, defined by Eq 2, between the channels for the above function *F*_*DFA*_: (c) Real and (d) Imaginary case. Here, Δ*logF*_32;37_ (∘), Δ*logF*_32;49_ (△), and Δ*logF*_32;54_ (*).

**Fig 4:**
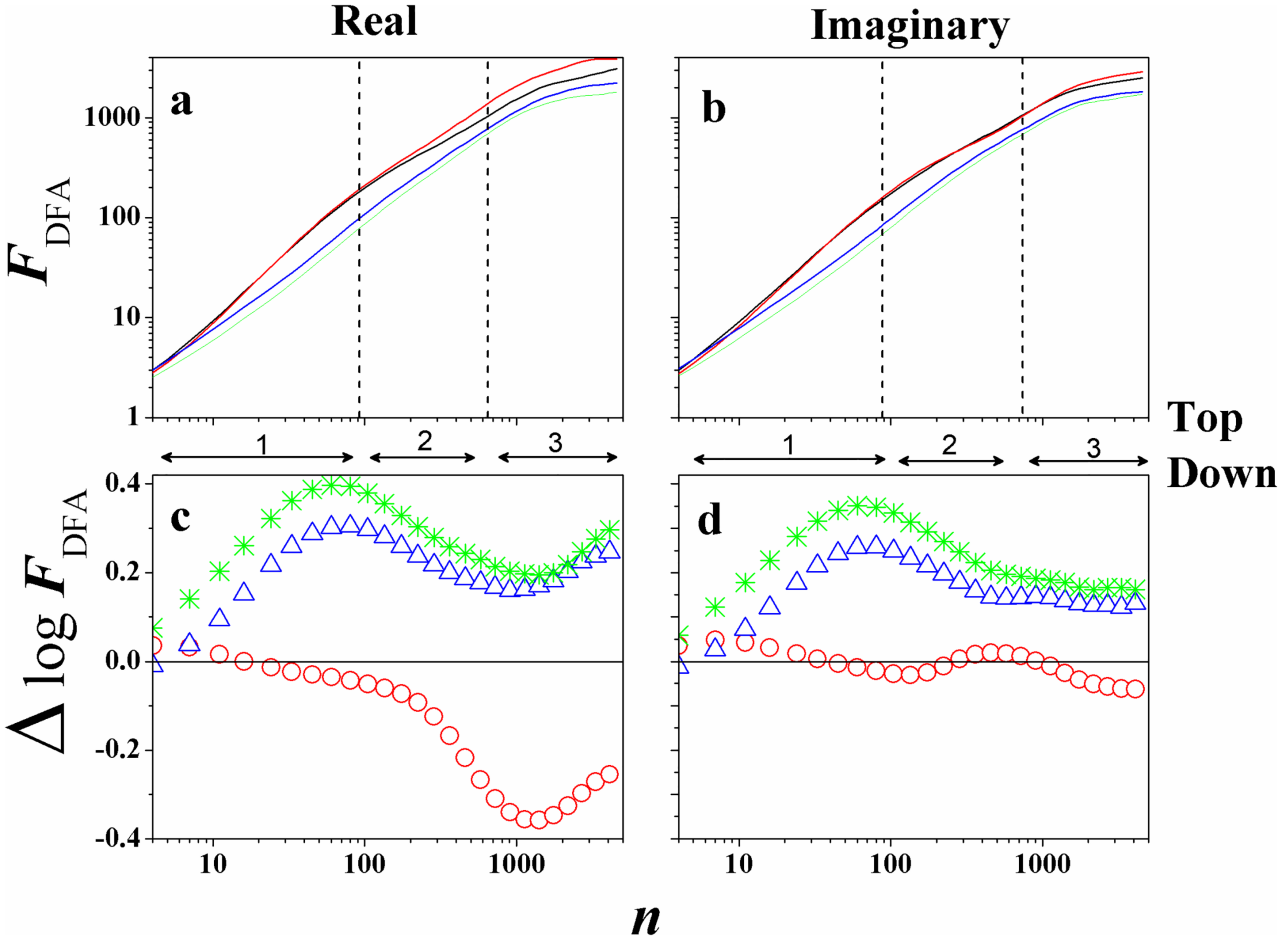
(Color on-line) *F*_*DFA*_ in function of *n* for *S*020 in the experiment 1 for (Top/Down): (a) Real and (b) Imaginary case. Black line represents *F*_3_32, red line *F*_6_37, blue line *P*_3_49, and green line *P*_6_54. Also, the figures below show the difference Δ*logF*_32;*xx*_, defined by Eq 2, between the channels for the above function *F*_*DFA*_: (c) Real and (d) Imaginary case. Here, Δ*logF*_32;37_ (∘), Δ*logF*_32;49_ (△), and Δ*logF*_32;54_ (*).

One motivation for estimating *α*_*DFA*_, or the root mean square fluctuation *F*_*DFA*_(*n*), lies in the fact that these measures may potentially be used to classify or discriminate between different types of EEG signals, like we can see in Fig 2. Thus, characterized by the fact that at specific time scales the signal have the same type of behavior (self-affinity, see Figs 3 and 4), we can define (crossing these behaviors) the limit of the variability transition in the EEG signals [25]. Anyway, *F*_*DFA*_(*n*) was conceived as a method for detrending local variability in a sequence of events, and hence providing insight into long-term variations in the data sets. With the DFA method it is possible to remove trends that often exist in the EEG, and estimate the scaling from a wider range.

## Results

Every time series of EEG motor/imaginary experience has approximately 2*min* (*N* ≅ 20,000 points) with Δ*t* = 0.00625*s*, for Task {1, 2, 3, 4} and three times repeated, see Table 1. Fig 2 presents an example of the original EEG signal in the Task 1 (a target appears on either the left or the right side of the screen. The subject opens and closes the corresponding fist until the target disappears. Then the subject relaxes. (Real (L/R))). In this figure, we can not see clearly which channels are the ones with the greatest amplitude, but with *F*_*DFA*_ it is simple and possible, as will see below.

For our analysis we selected randomly ten subjects from the Physionet on-line database: *S*020, *S*029, *S*043, *S*046, *S*050, *S*051, *S*060, *S*071, *S*086, and *S*099. After, we calculated *F*_*DFA*_ for every specific Task. Fig 3 (Real/Imaginary (L/R)) and Fig 4 (Real/Imaginary (T/D)) shows *F*_*DFA*_ × *n* (a and b) for all four Tasks and for *S*020, as an example,.

In the Figs 3 and 4 (c and d) we present a new function, defined as the difference *logF*_*DFA*_ between the channel *F*_3_32 with the others:

$$\begin{array}{c} \Delta logF_{32;xx}\equiv logF_{DFA_{-32}}-logF_{DFA_{-xx}} \end{array}$$ (2)

Therefore, Δ*logF*_32;*xx*_ give us information about the relative intensity of the rms fluctuation function, that is, if:

- Δ*logF*_32;*xx*_ > 0, the amplitude of the rms fluctuation function about the channel *F*_3_32, in relation of the channel *xx*, is larger;
- Δ*logF*_32;*xx*_ = 0, the amplitude of the rms fluctuation function about the channel *F*_3_32, in relation of the channel *xx*, is zero;
- Δ*logF*_32;*xx*_ < 0, the amplitude of the rms fluctuation function about the channel *F*_3_32, in relation of the channel *xx*, is smaller.

Now, taking into account all experiments, tasks, and subjects, initially as a result we calculated the *F*_*DFA*_, observing if there is or not a power-law $F\left(n\right)∼n^{\alpha_{DFA}}$ for these EEG time series. We observe that *F*_*DFA*_(*n*) does not appear as a single power-law (see [25]), but we can identify three behaviors (slope in the time scale range), like *F*_*DFA*_(*n*) ∝ *n*^*α*(*i*)^, with *i* = 1, 2, 3 (see Figs 3 and 4 vertical lines), where:

- i = **1**, 4 ≤ *n* ≤ 90, with *α*_1_;
- i = **2**, 91 ≤ *n* ≤ 655, with *α*_2_;
- i = **3**, *n* > 655, with *α*_3_.

For example, one visible transition is around *n* = 656 (*t* = 4.1*s*), corresponding here to a time between two rests in the experiments.

In possession of this information for every subject in their four Tasks, in all three experiments, we calculated the mean value of *α*’*s* (in a specific time scale) for the channels *F*_3_32, *F*_6_37, *P*_3_49, and *P*_6_54, and we place these values at the Table 3.

**Table 3:** Mean values of *α*_*DFA*_ for all experiments (three). First column represents the subjects, and their respective Task. The remaining columns represents the analyzed channels. Time scale represent the range for DFA analysis of *α*_*DFA*_(*n*) (slope): *α*_1_ in time scale **1**, *α*_2_ in time scale **2**, and *α*_3_ in time scale **3**. Last line show the mean value of the columns for: Real (L/R), Imag (L/R), Real (T/D), Imag (T/D).

Channel		32			37			49			54		
Time scale		1	2	3	1	2	3	1	2	3	1	2	3
20	Real (L/R)	1.36	0.90	0.45	1.40	0.97	0.49	1.12	1.02	0.41	1.14	1.21	0.47
	Imag (L/R)	1.00	0.87	0.50	1.35	0.87	0.50	1.08	1.10	0.52	1.10	1.09	0.43
	Real (T/D)	1.33	1.16	0.43	1.39	1.18	0.46	1.15	1.29	0.48	1.18	1.35	0.49
	Imag (T/D)	1.35	1.01	0.54	1.40	1.27	0.51	1.08	1.04	0.46	1.08	1.22	0.59
29	Real (L/R)	0.97	1.31	0.31	1.33	1.31	0.33	0.97	1.31	0.31	1.14	1.31	0.31
	Imag (L/R)	1.13	1.19	0.33	0.97	0.97	0.40	0.97	1.19	0.40	0.89	1.19	0.40
	Real (T/D)	1.18	1.14	0.24	1.07	1.19	0.45	1.02	1.37	0.32	0.95	1.39	0.29
	Imag (T/D)	0.95	1.30	0.24	0.95	0.99	0.24	0.95	1.39	0.24	0.95	1.39	0.24
43	Real (L/R)	1.09	1.08	0.65	0.96	0.93	0.96	1.09	1.17	0.65	1.07	1.08	0.56
	Imag (L/R)	1.29	0.94	0.44	1.29	0.94	0.44	0.96	1.12	0.60	0.87	1.08	0.58
	Real (T/D)	1.29	0.99	0.48	1.21	0.99	0.45	1.05	1.12	0.48	1.05	1.11	0.49
	Imag (T/D)	1.11	0.97	0.49	0.98	0.97	0.41	1.11	1.17	0.49	1.06	1.06	0.46
46	Real (L/R)	1.18	0.97	0.56	1.18	1.01	0.56	1.18	1.01	0.56	0.93	1.01	0.56
	Imag (L/R)	1.14	0.91	0.35	1.14	0.91	0.35	1.09	1.04	0.32	1.01	1.01	0.56
	Real (T/D)	1.26	0.90	0.40	1.26	0.90	0.40	1.01	1.02	0.39	1.01	1.02	0.39
	Imag (T/D)	1.29	0.74	0.43	1.29	0.74	0.43	1.17	0.88	0.49	1.17	0.88	0.49
50	Real (L/R)	1.10	0.81	0.48	0.97	0.81	0.43	0.94	1.01	0.51	0.95	0.88	0.50
	Imag (L/R)	0.98	0.99	0.55	0.86	0.94	0.45	0.90	1.01	0.55	0.92	0.84	0.52
	Real (T/D)	1.01	0.89	0.43	0.90	0.91	0.48	0.89	0.94	0.49	0.90	0.81	0.52
	Imag (T/D)	1.08	0.85	0.41	0.94	0.88	0.38	0.94	0.97	0.43	0.95	0.89	0.45
51	Real (L/R)	1.40	0.82	0.40	1.44	0.86	0.38	1.16	0.95	0.65	1.17	0.96	0.53
	Imag (L/R)	1.41	0.80	0.34	1.46	0.80	0.38	1.19	0.97	0.57	1.20	1.01	0.55
	Real (T/D)	1.44	0.81	0.37	1.47	0.79	0.36	1.21	0.95	0.60	1.21	0.96	0.59
	Imag (T/D)	1.45	0.77	0.58	1.49	0.76	0.54	1.25	0.99	0.82	1.23	1.01	0.91
60	Real (L/R)	1.22	1.01	0.69	1.19	0.92	0.68	0.99	1.18	0.85	1.01	1.21	0.90
	Imag (L/R)	1.12	1.08	0.86	1.09	0.96	0.76	0.92	1.26	0.89	0.94	1.32	0.91
	Real (T/D)	1.06	1.01	0.75	1.08	0.92	0.73	0.86	1.22	0.80	0.85	1.30	0.85
	Imag (T/D)	1.16	1.08	0.70	1.16	0.96	0.70	0.90	1.21	0.79	0.94	1.25	0.85
71	Real (L/R)	0.94	1.05	0.54	0.94	1.08	0.51	0.88	1.06	0.55	0.93	1.07	0.57
	Imag (L/R)	0.94	1.13	0.65	0.93	1.07	0.51	0.89	1.03	0.55	0.93	1.04	0.59
	Real (T/D)	0.87	1.08	0.61	0.93	1.07	0.52	0.92	1.03	0.52	0.92	1.08	0.58
	Imag (T/D)	0.93	1.05	0.94	0.94	1.07	0.50	0.88	1.06	0.55	0.93	1.06	0.56
86	Real (L/R)	1.36	0.88	0.50	1.39	0.81	0.53	1.15	0.98	0.57	1.15	1.03	0.49
	Imag (L/R)	1.38	0.75	0.49	1.39	0.77	0.48	1.19	0.87	0.48	1.21	0.91	0.44
	Real (T/D)	1.36	0.87	0.48	1.39	0.88	0.53	1.14	1.04	0.49	1.17	1.06	0.48
	Imag (T/D)	1.36	0.85	0.46	1.37	0.84	0.50	1.17	0.96	0.49	1.18	0.96	0.51
99	Real (L/R)	0.82	0.87	0.18	0.92	0.87	0.18	0.79	1.12	0.21	0.82	1.30	0.18
	Imag (L/R)	0.84	0.88	0.21	0.99	0.86	0.19	0.79	1.08	0.18	0.82	1.27	0.15
	Real (T/D)	0.84	0.80	0.25	0.98	0.85	0.17	0.81	0.96	0.22	0.81	1.19	0.19
	Imag (T/D)	0.90	0.78	0.18	1.04	0.77	0.25	0.82	0.97	0.23	0.85	1.21	0.17
mean	Real (L/R)	1.14	0.97	0.48	1.17	0.96	0.51	1.03	1.08	0.53	1.03	1.11	0.51
	Imag (L/R)	1.12	0.95	0.47	1.15	0.91	0.45	1.00	1.07	0.51	0.99	1.08	0.51
	Real (T/D)	1.16	0.97	0.44	1.17	0.97	0.46	1.01	1.09	0.48	1.01	1.13	0.49
	Imag (T/D)	1.16	0.94	0.50	1.16	0.93	0.45	1.03	1.06	0.50	1.03	1.09	0.52

At the end of the Table 3 we present the global average value of *α*_*DFA*_ for the following Task:(Real (Left/Right)), (Imag (Left/Right)), (Real (Top/Down)), and (Imag (Top/Down)). These exponents clearly are time dependent, with specific value. For example:

- time scale **1** has *α*_*DFA*_ > 1 (representing a non-stationary case);
- time scale **2** has *α*_*DFA*_ ≃ 1 (representing a 1/*f* noise) and;
- time scale **3** has *α_DFA_* ⋍ 0.5 (representing a random case).

**Fig 5:**
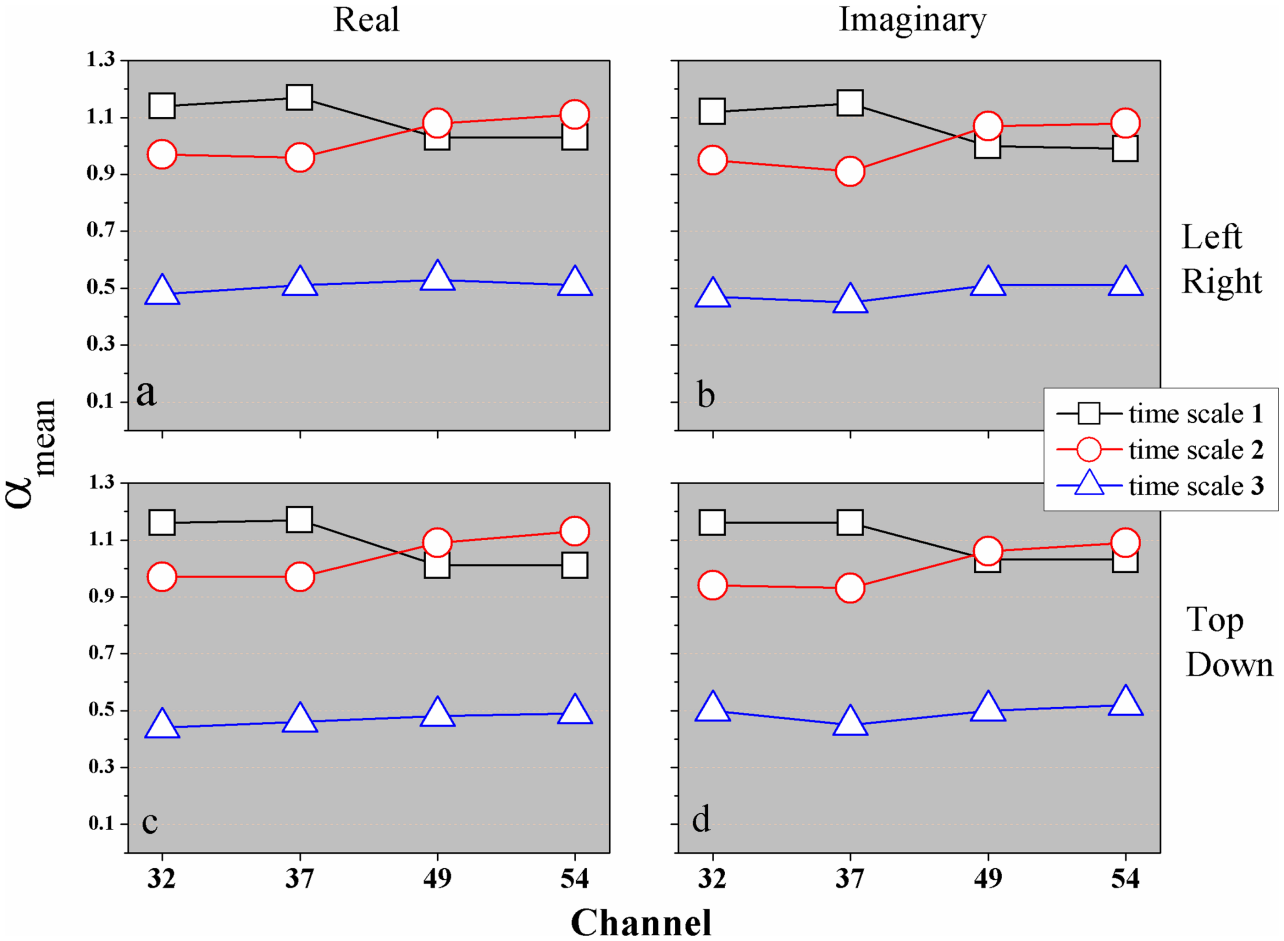
(Color on-line) Mean values of *α*_*DFA*_ for all subjects in all Tasks: (a) Real (L/R), (b) Imag (L/R), (c) Real (T/D), and (d) Imag (T/D) Tasks. The symbol (□) represents time scale **1**, (○) time scale **2**, and (△) time scale **3**.

We noticed that, *α*_*DFA*_ is independent of the Task performed by the subject, see Fig 5 for better visualization.

However, our main objective was to measure the Δ*logF*_32;*xx*_, to compare the brain activities between the hemispheres (left/right and frontal/parietal). Figs 3 and 4 showed preliminary results of this study, with Δ*logF*_32;*xx*_ in function of *n*, and interesting things can be observed. We can identify that:

$$\begin{array}{ccc} \Delta logF_{32;49} & > & 0,\\ \Delta logF_{32;54} & > & 0,\text{and}\\ \Delta logF_{32;37} & ≃ & 0. \end{array}$$

**Fig 6:**
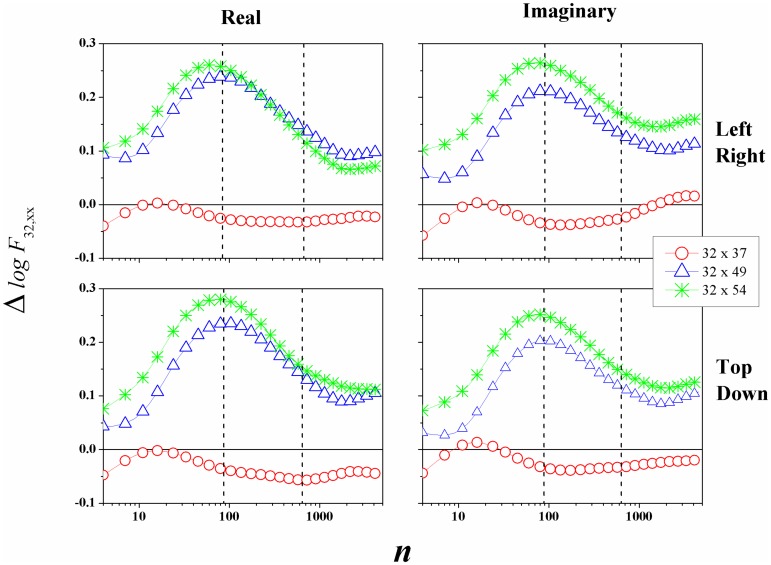
(Color on-line) Mean value of Δ*logF*_32;*xx*_ between the channel *F*_3_32 and others three. Every curve represent the difference between *F*_3_32 and: (○) *F*_6_37, (△) *P*_3_49, and (*) *P*_6_54.

This analysis shows the greater prevalence in amplitude of the frontal channels in relation to the parietal channels, for this Task. The maximum of Δ*logF*_32;49_, Δ*logF*_32;54_ is found in *n* ≃ 90 (*t* = 0.56*s*), and the Fig 6 presents the global average of the rms fluctuation function.

If we remember what means Δ*logF*_32;*xx*_, Eq 2, then we clearly see that the channels *P*_3_49 and *P*_6_54 have a smaller fluctuation if we compare with channel *F*_3_32, and the maximum of this difference is around *n* = 90 (*t* = 0.56*s*). The difference between *F*_3_32 and *F*_6_37 is approximately zero, but with a slight negative level. This result shows that the channel *F*_6_37 has a higher value for *F*_*DFA*_ rms function. But, for *n* ≃ 15 the amplitude in relation to channel *F*_3_32 had the same magnitude, because Δ*logF*_32;37_ ≃ 0.

## Discussion

In this paper we studied how the amplitude of the rms fluctuation function, *F*_*DFA*_, behaves in a 64 channels EEG, taken for 10 subject in different tasks (motor/imaginary). In this sense, in a logical way, we analyzed how the cerebral hemispheres left/right (frontal) and left/right (parietal) are related. As you know, the left side of the brain is responsible for controlling the right side of the body. It also performs tasks that have to do with logic. On the other hand, the right hemisphere coordinates the left side of the body, and performs tasks that have to do with creativity. Already the parietal lobe, integrates sensory information, including spacial sense and navigation. The major sensory inputs from the skin (touch, temperature, and pain receptors), relay through the thalamus to the parietal lobe. Also, areas of the parietal lobe are important in language processing.

Thus, by the motor/imaginary experience presented here, where the subjects perform activities that are not related with the parietal lobe, we expect to find a greater activity in the frontal channels. And even more, how does this happen in time scale? In this sense we chose the central channels in each hemisphere (frontal and parietal), present here by *F*_3_32 (left frontal), *F*_6_37 (right frontal), *P*_3_49 (left parietal), and *P*_6_54 (right parietal). Hence, applying the DFA method in first hand, we found three time scales with three values of *α*_*DFA*_ exponents, see Table 3 and Fig 5. This figure shows that the channels *F*_3_32 and *F*_6_37 behave in a similar way, in other words, for time scale **1** the tendency is to find *α*_*DFA*_ > 1 (non-stationary) and for time scale **2** the value of *α*_*DFA*_ ≃ 1 (1/*f* noise). For time scale **3** *α_DFA_* ⋍ 0.5 (uncorrelated). However, this situation changes for the channels *P*_3_49 and *P*_6_54, because time scales **1** and **2** tend to a same value for *α*_*DFA*_, mainly the channel *P*_3_49.

We provide that the current manuscript advances on previous work, because the EEG signal is mostly analyzed in the frequency domain and here, with DFA method, we are analyzing the EEG signal in the time domain, which allows us to see directly the time scale. Thus, with the auto-correlation exponent (*α*_*DFA*_), we could identify three time scales for the rms fluctuation function, that are: i) Time scale **1**, with 4 ≤ *n* ≤ 90; ii) Time scale **2**, with 91 ≤ *n* ≤ 655; iii) Time scale **3**, with *n* > 655. Also, we can compare the EEG channels by rms function, and infer which channel has the greatest (or not) amplitude (brain activity). This goal was reached when we defined Eq 2 and obtained the results of EEG time series. In our analysis, the frontal channels are the ones that present greater fluctuation in the Motor/Imaginary activities, if we compared to the parietal channels. This is a new way to analyze the EEG signals, because it has not yet been implemented, and that may help in the future assist EEG analysis of people with some type of brain disorder.

We can see in these results that the proposed method can be used to interpret the functioning of the brain from the point of view of the DFA functional mapping, during motor activation in real/imaginary situation.

## Conclusion

In this paper we propose a new methodology to analyze EEG signals, which are generally treated in the frequency spectrum, by Fourier for example. We study the *F*_*DFA*_ rms function. Therefore, analyzing the channels *F*_3_32, *F*_6_37 (frontal region of the head), *P*_3_49, and *P*_6_54 (parietal region of the head), we found that the amplitude of fluctuation tends to be larger in the frontal channels (*F*_3_32 and *F*_6_37), if we compare with the channels located in the parietal region of the brain (*P*_3_49 and *P*_6_54).

We start this paper by calculating the auto-correlation exponent *α*_*DFA*_, that show three values *α*_1_ (time scale **1**), *α*_2_ (time scale **2**), and *α*_3_ (time scale **3**). For *F*_3_32 and *F*_6_37 the auto-correlation exponent *α*_1_ > *α*_2_. But, for *P*_3_49 and *P*_6_54, *α*_1_ < *α*_2_. For large time scales, *n* > 656 or *t* > 4.1*s* (which corresponds to the interval between two rests) the time series of EEG human motor/imaginary has a same type of behavior for all Tasks. In this time scale **3** *α*_3_ ≃ 0.5 (uncorrelated time series), for all Tasks (real/imaginary, left/right, top/down) and channels (see Fig 5).

Our goal was that, from *F*_*DFA*_ and the information about the EEG channels amplitude, we define Δ*logF*_32;*xx*_, and we applied this new function for human EEG motor/imaginary analysis. In this sense, we did not identify in this analysis large differences between motor/imaginary activity, except for the small difference between real/imaginary and left/right Tasks at the channels *P*_3_49 and *P*_6_54, because Δ*logF*_32;49_ < Δ*logF*_32;54_ Fig 6. Likewise, we identify a peak in Δ*logF*_32;*xx*_ located at *n* ≃ 90 (*t* = 0.56*s*). For *n* > 656 (*t* > 4.1*s*), Δ*logF*_32;*xx*_ tends to a constant value.

In order to improve the statistics, we also calculated the difference, Δ*logF*_32;*xx*_, between the channel 32 and the channel 09, 11, and 13 (center of the brain, see Fig 1). The results (not shown here) are very similar to those found between the channel 32 and those below (parietal region). Also, for test the reference channel in our raw data, we considered as a reference electrode standardization technique (REST) [26, 27]. The results of Δ*logF*_32;*xx*_(*rest*) are qualitatively similar for the original time series, changing only in the amplitude, smaller in the REST.

Finally, this analysis could be done taking into account a single individual (such as *S*020 explained above). In this case, Δ*logF*_*yy*;*xx*_ analysis can be very useful for comparing channels (*yy*;*xx*) in individuals with some type of anomaly, such as seizures, epilepsy, head injuries, dizziness, headaches, brain tumors and sleeping problems, amongst others. This is a novel strategy to study brain activity in EEG.

## Supporting information

S1 Table Values of *α*_*DFA*_ for all subjects. First column represents the subjects, and their respective Task. The remaining columns represents the analyzed channels. Results for Channels *C*_3_9, *C*_*z*_11, and *C*_4_13 (central part of the brain).
(PDF)

S1 Fig Mean values of *α*_*DFA*_ exponents for all subjects in all Tasks. (a) Real (L/R), (b) Imag (L/R), (c) Real (T/D), and (d) Imag (T/D). Results for Channels *C*_3_9, *C*_*z*_11, and *C*_4_13 (central part of the brain).
(PDF)

S2 Fig *F*_*DFA*_ in function of *n* for *S*020 in the experiment 1, the below figure show the difference defined by Eq 2. Here we have a Left/Right case. Results for Channels *C*_3_9, *C*_*z*_11, and *C*_4_13 (central part of the brain).
(PDF)

S3 Fig *F*_*DFA*_ in function of *n* for *S*020 in the experiment 1, the below figure show the difference defined by Eq 2. Here we have a Top/Down case. Results for Channels *C*_3_9, *C*_*z*_11, and *C*_4_13 (central part of the brain).
(PDF)

## References

[1] Wikiwand: Available from: http://www.wikiwand.com/en/Electroencephalography.

[2] William O. Tatum IV. Handbook of EEG interpretation. Second edition ed. ISBN: 978-1-62070-016-7. Demos Medical Publishing; 2014.

[3] Yang S. The use of EEG signals for biometric person recognition. University of Kent; 2015. Available from: https://kar.kent.ac.uk/53681/1/235Thesis%20(Su%20Yang).pdf.

[4] Swartz BE. The advantages of digital over analog recording techniques. Electroencephalography and Clinical Neurophysiology. 1998;106(2):113–117. 10.1016/S0013-4694(97)00113-2 9741771

[5] Wolpaw JR, Wolpaw EW. Brain-Computer Interfaces: Principles and Practice. Oxford University Press, New York; 2012.

[6] Goldberger AL, Amaral LAN, Glass L, Hausdorff JM, Ivanov PC, Mark RG, et al PhysioBank, PhysioToolkit, and PhysioNet: Components of a New Research Resource for Complex Physiologic Signals. Circulation. 2000;101(23):e215–e220. 10.1161/01.CIR.101.23.e215 10851218

[7] Schalk G, McFarland DJ, Hinterberger T, Birbaumer N, Wolpaw JR. BCI2000: a general-purpose brain-computer interface (BCI) system. IEEE Transactions on Biomedical Engineering. 2004;51(6):1034–1043. 10.1109/TBME.2004.827072 15188875

[8] BCI2000: a general-purpose brain-computer interface (BCI) system; 2004. Available from: http://www.schalklab.org/publications/2004/bci2000-general-purpose-brain-computer-interface-bci-system. 10.1109/TBME.2004.827072 15188875

[9] EEG Motor Movement/Imagery Dataset;. Available from: http://physionet.fri.uni-lj.si/physiobank/database/eegmmidb/HEADER.shtml.

[10] Curtis CE, D’Esposito M. Persistent activity in the prefrontal cortex during working memory. Trends Cogn Sci. 2003;7(9):415–423. 10.1016/S1364-6613(03)00197-9 12963473

[11] Romine CB, Reynolds CR. Sequential memory: a developmental perspective on its relation to frontal lobe functioning. Neuropsychol Rev. 2004;14(1):43–64. 10.1023/B:NERV.0000026648.94811.32 15260138

[12] Peng CK, Buldyrev SV, Havlin S, Simons M, Stanley HE, Goldberger AL. Mosaic organization of DNA nucleotides. Phys Rev E. 1994;49:1685–1689. 10.1103/PhysRevE.49.1685 9961383

[13] Walleczek J. Self-organized Biological Dynamics and Nonlinear Control. Cambridge University Press, UK; 2000.

[14] Heneghan C, McDarby G. Establishing the relation between detrended fluctuation analysis and power spectral density analysis for stochastic processes. Phys Rev E. 2000;62(5):6103–6110. 10.1103/PhysRevE.62.6103 11101940

[15] Mirzayof D, Ashkenazy Y. Preservation of long range temporal correlations under extreme random dilution. Physica A. 2010;389:5573–5580. 10.1016/j.physa.2010.08.035

[16] Hurst H. Long Term Storage Capacity of Reservoirs. Transactions of the American Society of Civil Engineers. 1951;116:770–799.

[17] Chen Z, Ivanov PC, Hu K, Stanley HE. Effect of nonstationarities on detrended fluctuation analysis. Phys Rev E. 2002;65:041107 10.1103/PhysRevE.65.041107 12005806

[18] Hu K, Ivanov PC, Chen Z, Carpena P, Eugene Stanley H. Effect of trends on detrended fluctuation analysis. Phys Rev E. 2001;64:011114 10.1103/PhysRevE.64.011114 11461232

[19] Márton LF, Brassai ST, Bakó L, Losonczi L. Detrended Fluctuation Analysis of EEG Signals. Procedia Technology. 2014;12:125–132. 10.1016/j.protcy.2013.12.465

[20] Lee JS, Yang BH, Lee JH, Choi JH, Choi IG, Kim SB. Detrended fluctuation analysis of resting EEG in depressed outpatients and healthy controls. Clinical Neurophysiology. 2017;118(11):2489–2496. 10.1016/j.clinph.2007.08.001 17890151

[21] Hardstone R, Poil SS, Schiavone G, Jansen R, Nikulin V, Mansvelder H, et al Detrended Fluctuation Analysis: A Scale-Free View on Neuronal Oscillations. Frontiers in Physiology. 2012;3:450 10.3389/fphys.2012.00450 23226132 PMC3510427

[22] Lee JM, AU Kim DJ, AU Kim IY, AU Park KS, AU Kim SI. Detrended fluctuation analysis of EEG in sleep apnea using MIT/BIH polysomnography data. Computers in Biology and Medicine. 2002;32(1):37–47. 10.1016/S0010-4825(01)00031-2 11738639

[23] Kantelhardt JW, Zschiegner SA, Koscielny-Bunde E, Havlin S, Bunde A, Stanley HE. Multifractal detrended fluctuation analysis of nonstationary time series. Physica A: Statistical Mechanics and its Applications. 2002;316(1–4):87–114. 10.1016/S0378-4371(02)01383-3

[24] Galaska R, Makowiec D, Dudkowska A, Koprowski A, Fijalkowski M, Wdowczyk-Szulc J, et al Multifractal properties of heart rate by multifractal detrended fluctuation analysis and wavelet transform modulus maxima analysis—are both approaches equivalent? Journal of Electrocardiology. 2007;40(4):S41 10.1016/j.jelectrocard.2007.03.404

[25] Zebende GF, Fernandez BF, Pereira MG. Analysis of the variability in the sdB star KIC 10670103: DFA approach. Mon Not R Astron Soc. 2017;464(3):2638–2642. 10.1093/mnras/stw2611

[26] Yao D. A method to standardize a reference of scalp EEG recordings to a point at infinity. Physiol Meas. 2001;22(4):693–711. 10.1088/0967-3334/22/4/305 11761077

[27] Yao D. Reference Electrode Standardization Technique;. Available from: http://www.neuro.uestc.edu.cn/rest/.

