# Supplementary material for: Auto-correlation in the motor/imaginary human EEG signals: A vision about the FDFA fluctuations
Source: PLoS One. 2017 Sep 14;12(9):e0183121. doi: 10.1371/journal.pone.0183121 (PMC5598924; doi:10.1371/journal.pone.0183121)
Supplement: S1 Table — First column represents the subjects, and their respective Task. The remaining columns represents the analyzed channels. Results for Channels C39, Cz11, and C413 (central part of the brain). (PDF) [file pone.0183121.s001.pdf]

DFA exponents for all subjects.

Channel		3			13			11		
time scale		1	2	3	1	2	3	1	2	3
20	Real (L/R)	1.18	0.96	0.46	1.16	0.97	0.43	1.18	0.97	0.44
	Imag (L/R)	1.44	0.96	0.54	1.12	0.95	0.53	1.13	0.98	0.47
	Real (T/D)	1.44	0.96	0.54	1.12	0.95	0.53	1.13	0.98	0.47
	Imag (T/D)	1.40	0.96	0.52	1.13	0.97	0.49	0.98	0.97	0.59
29	Real (L/R)	1.07	1.26	0.29	1.07	1.26	0.29	1.07	1.26	0.29
	Imag (L/R)	0.93	1.27	0.28	0.93	1.27	0.28	0.93	1.27	0.28
	Real (T/D)	0.93	1.27	0.28	0.93	1.27	0.28	0.93	1.27	0.28
	Imag (T/D)	1.07	1.26	0.29	1.07	1.26	0.29	1.07	1.26	0.29
43	Real (L/R)	1.07	0.98	0.60	1.07	0.98	0.52	1.07	0.98	0.52
	Imag (L/R)	0.96	1.02	0.54	1.14	1.09	0.47	1.14	0.98	0.52
	Real (T/D)	1.05	1.07	0.56	1.09	1.08	0.58	1.05	1.07	0.56
	Imag (T/D)	0.96	1.02	0.54	1.14	1.09	0.47	1.14	0.98	0.52
46	Real (L/R)	1.14	0.99	0.51	1.29	0.92	0.48	1.16	0.99	0.44
	Imag (L/R)	0.89	1.02	0.40	1.03	0.99	0.24	1.03	1.00	0.29
	Real (T/D)	0.89	0.85	0.49	1.17	0.86	0.45	1.17	0.86	0.45
	Imag (T/D)	0.90	1.00	0.45	1.04	0.96	0.36	1.04	0.96	0.36
50	Real (L/R)	1.03	0.88	0.50	1.03	0.88	0.50	1.03	0.88	0.57
	Imag (L/R)	0.89	0.97	0.48	0.95	0.89	0.49	0.92	1.04	0.54
	Real (T/D)	0.87	0.91	0.45	0.87	0.91	0.45	0.87	0.91	0.45
	Imag (T/D)	0.88	0.91	0.47	1.02	0.89	0.51	0.94	0.90	0.57
51	Real (L/R)	1.02	0.94	0.54	1.01	0.92	0.53	1.01	0.95	0.54
	Imag (L/R)	0.89	0.94	0.48	1.01	0.89	0.52	1.00	0.99	0.58
	Real (T/D)	0.90	0.88	0.50	0.90	0.89	0.51	0.90	0.88	0.51
	Imag (T/D)	0.90	0.88	0.48	0.90	0.89	0.50	0.90	0.89	0.49
60	Real (L/R)	1.05	1.12	0.90	1.05	1.12	0.88	1.03	1.12	0.88
	Imag (L/R)	1.08	1.09	1.08	1.08	1.01	0.88	1.08	1.07	0.82
	Real (T/D)	1.03	1.07	0.80	1.02	1.03	0.64	1.00	1.04	0.64
	Imag (T/D)	0.99	0.85	0.67	1.20	0.77	0.54	1.15	0.99	0.76
71	Real (L/R)	0.99	1.21	0.70	1.21	0.92	0.72	0.92	1.29	0.63
	Imag (L/R)	1.07	1.22	0.70	0.95	1.18	0.60	0.93	1.23	0.58
	Real (T/D)	1.10	1.24	0.72	0.95	1.18	0.59	0.93	1.24	0.60
	Imag (T/D)	1.06	1.23	0.63	0.93	1.20	0.63	0.91	1.21	0.60
86	Real (L/R)	1.24	0.86	0.50	1.26	0.88	0.48	1.24	0.90	0.47
	Imag (L/R)	1.28	0.78	0.46	1.30	0.78	0.39	1.28	0.81	0.47
	Real (T/D)	1.24	0.86	0.50	1.26	0.88	0.48	1.24	0.90	0.47
	Imag (T/D)	1.28	0.81	0.47	1.27	0.94	0.43	1.24	0.93	0.43
99	Real (L/R)	0.80	0.92	0.20	0.82	1.03	0.18	0.78	0.93	0.18
	Imag (L/R)	0.72	0.90	0.13	0.84	0.99	0.14	0.78	0.96	0.21
	Real (T/D)	0.69	0.78	0.14	0.87	0.87	0.18	0.81	0.82	0.17
	Imag (T/D)	0.67	0.78	0.17	0.83	0.90	0.19	0.80	0.83	0.20
mean	Real(L/R)	1.06	1.01	0.52	1.10	0.99	0.50	1.05	1.03	0.50
	Imag(L/R)	1.02	1.02	0.51	1.04	1.00	0.45	1.02	1.03	0.48
	Real(T/D)	1.01	0.99	0.50	1.02	0.99	0.47	1.00	1.00	0.46
	Imag(T/D)	1.01	0.97	0.47	1.05	0.99	0.44	1.02	0.99	0.48
