# Supplementary material for: Auto-correlation in the motor/imaginary human EEG signals: A vision about the FDFA fluctuations
Source: PLoS One. 2017 Sep 14;12(9):e0183121. doi: 10.1371/journal.pone.0183121 (PMC5598924; doi:10.1371/journal.pone.0183121)
Supplement: S1 Fig — (a) Real (L/R), (b) Imag (L/R), (c) Real (T/D), and (d) Imag (T/D). Results for Channels C39, Cz11, and C413 (central part of the brain). (PDF) [file pone.0183121.s002.pdf]

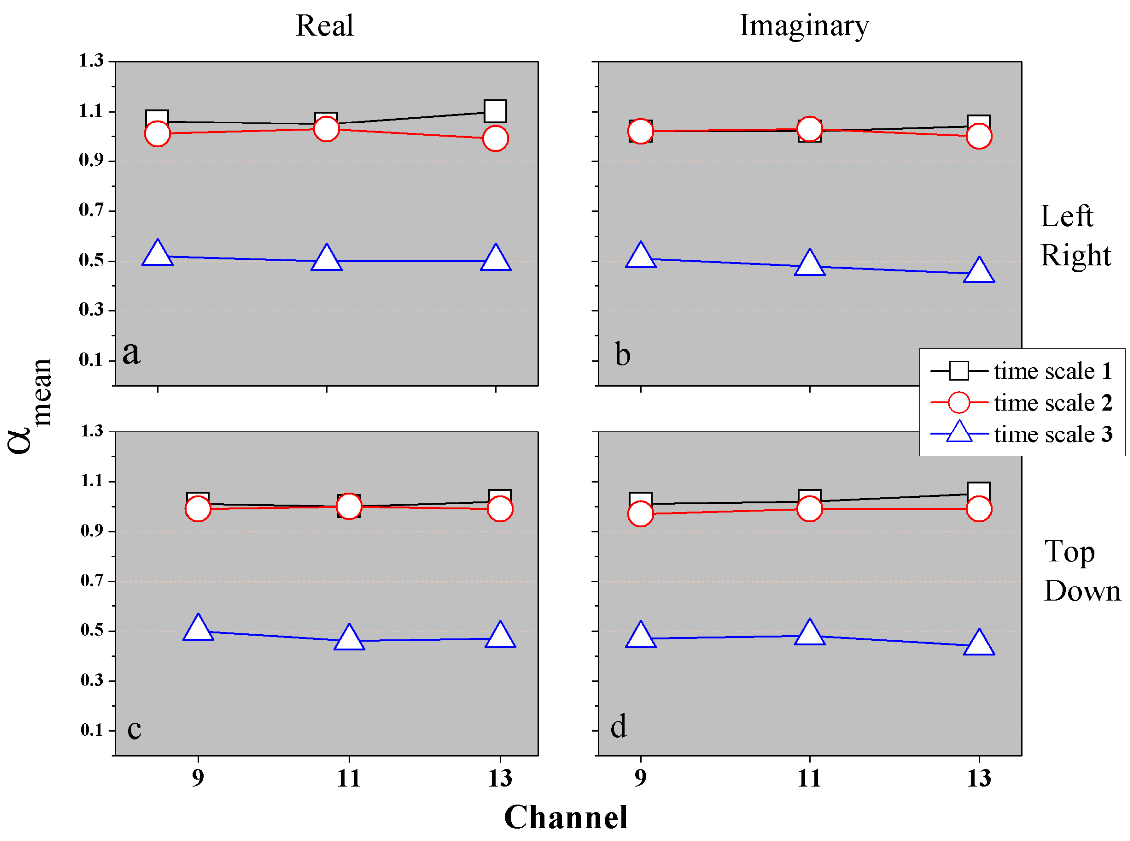

(Color on-line) Mean values of DFA exponents for all subjects in all Tasks: (a) Real (L/R), (b) Imag (L/R), (c) Real (T/D), and (d) Imag (T/D).
