# Supplementary material for: Auto-correlation in the motor/imaginary human EEG signals: A vision about the FDFA fluctuations
Source: PLoS One. 2017 Sep 14;12(9):e0183121. doi: 10.1371/journal.pone.0183121 (PMC5598924; doi:10.1371/journal.pone.0183121)
Supplement: S3 Fig — Here we have a Top/Down case. Results for Channels C39, Cz11, and C413 (central part of the brain). (PDF) [file pone.0183121.s004.pdf]

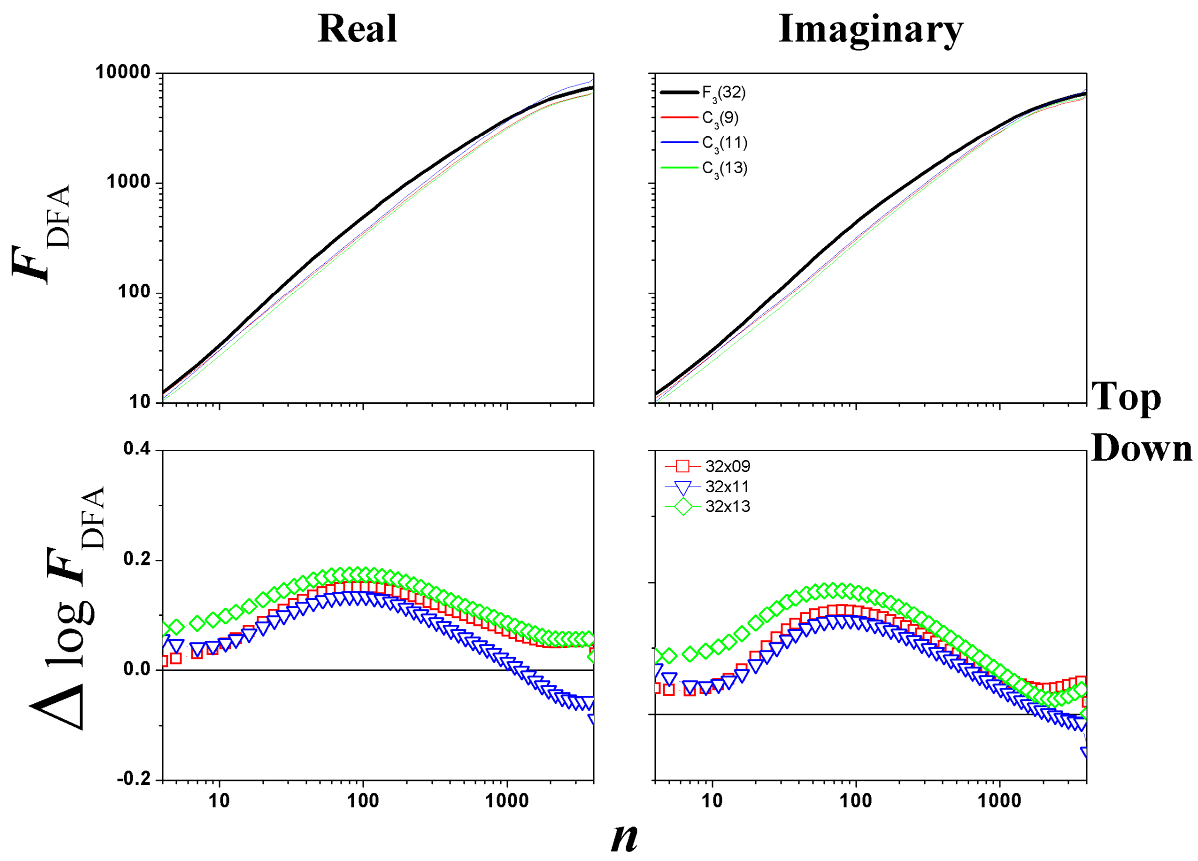

(Color on-line) FDFA in function of n for S020 in the experiment 1 for (Top/Down): (a) Real and (b) Imaginary case. The figures below show the difference defined by Equation 2, between the channels for the above function FDFA:(c) Real and (d) Imaginary case.
